# A Simple Method to Quantitate IP-10 in Dried Blood and Plasma Spots

**DOI:** 10.1371/journal.pone.0039228

**Published:** 2012-06-27

**Authors:** Martine G. Aabye, Jesper Eugen-Olsen, Anne Marie Werlinrud, Line Lindebo Holm, Tamara Tuuminen, Pernille Ravn, Morten Ruhwald

**Affiliations:** 1 Clinical Research Centre, Copenhagen University Hospital, Hvidovre, Denmark; 2 Department of Internal Medicine, Infectious Diseases Unit, Herlev University Hospital, Copenhagen, Denmark; 3 Department of Bacteriology and Immunology, Haartman Institute, University of Helsinki, Helsinki, Finland; 4 Department of Infectious Diseases, Odense University Hospital, Odense, Denmark; University of Cape Town, South Africa

## Abstract

**Background:**

Antigen specific release of IP-10 is an established marker for infection with *M.tuberculosis*. Compared to IFN-γ, IP-10 is released in 100-fold higher concentrations enabling the development of novel assays for detection. Dried blood spots are a convenient sample for high throughput newborn screening.

**Aim:**

To develop a robust and sensitive ELISA-based assay for IP-10 detection in plasma, dried blood spots (DBS) and dried plasma spots (DPS); to validate the ELISA in clinically relevant samples; and to assess the performance of the assay for detection of Cytomegalovirus (CMV) and *M.tuberculosis* specific immune responses.

**Method:**

We raised mice and rat monoclonal antibodies against human IP-10 and developed an ELISA. The assay was validated and applied to the detection of CMV and *M.tuberculosis* specific responses in 18 patients with immune reactivity towards *M.tuberculosis* and 32 healthy controls of which 22 had immune reactivity towards CMV and none towards *M.tuberculosis*. We compared the performance of this new assay to IFN-γ.

**Results:**

The ELISA was reliable for IP-10 detection in both plasma and filter paper samples. The linear range of the ELISA was 2.5–600 pg/ml. IFN-γ was not readily detectable in DPS samples. IP-10 was stabile in filter paper samples for at least 4 weeks at 37°C. The correlation between IP-10 detected in plasma, DPS and DBS samples was excellent (r^2^>0.97).

**Conclusions:**

This newly developed assay is reliable for IP-10 quantification in plasma, DBS and DPS samples from antigen stimulated and non-stimulated whole blood. The filter paper assays enable easy sample acquisition and transport at ambient temperature e.g. via the postal system. The system can potentially simplify diagnostic assays for *M.tuberculosis* and CMV infection.

## Introduction

The chemokine IP-10 (CXCL-10) is a small 7.2 Kd pro-inflammatory molecule, expressed by different cells including monocyte/macrophages, hepatocytes, endothelial cells, fibroblasts and astrocytes. Once secreted, IP-10 is involved in trafficking of neutrophils and lymphocytes (CD4+ and CD8+ T cells, NK and NK T cells) to the sites of inflammation (Reviewed in [1], [2]). A series of recent studies suggest that IP-10 is a potential correlate of on-going inflammation [3]. Elevated IP-10 levels have been associated with infectious diseases, immune dysfunction and tumour development [1], [3]–[5], and can be used to monitor hepatitis C viral infection [6], [7], tuberculosis [8]–[11] and malaria [12].

IP-10 is an inducible cytokine, and expression is stimulated at the transcription levels by multiple signals, mainly T cell derived IFN-γ and TNF-α but also IL-2, type II IFNs, IL-27, IL-17/IL-23 and IL-1β. TNF-α is a weak IP-10 inducer *per-se*, but a potent synergistic inducer with the IFNs [1], [13]–[22]. IP-10 has emerged as a promising alternative read-out marker to IFN-γ in cell mediated immune response (CMI) assays e.g. in the Quantiferon test (QFT-TB, Qiagen, USA) used for diagnosis of infection with *M.tuberculosis* (rev. in [2]).

IP-10 is a downstream marker compared to IFN-γ and other classical T cell cytokines in CMI assays, which may explain recent reports suggesting that IP-10 is a more robust diagnostic marker than IFN-γ in young children and patients with advanced HIV infection [23]–[28]. Importantly, IP-10 is expressed in 100-fold higher levels than IFN-γ, which opens new possibilities for assay simplification and miniaturization e.g. lateral flow, dried blood spots and molecular detection [2], [5].

Blood and plasma spotted onto filter paper is a simple method for preserving and transport of blood borne analytes such as aminoacids, antibodies, viral nucleic acids, hormones and proteins [29]–[31]. Dr. Robert Guthrie developed the technique in the early 1960ies for neonatal screening for phenylketonuria, and routine screening has now been expanded to 25–50 disorders. Neonatal screening using DBS is standard of care worldwide [32], [33]. Filter paper technology is also used for viral disease management including HIV viral load monitoring and HIV screening in many countries including South Africa [31], [34], [35].

The optimal requirements for sample application, storage, elution and analysis of small molecules and metabolites from filter paper have been extensively studied [29], [36]. However, the clinical potential, and importantly, the assay conditions for the detection of blood cytokines from filter paper samples have attracted little attention [37], [38]. No assessment of IP-10 detection from filter paper has been done so far.

In this article we describe the development of an ELISA for IP-10 detection in plasma, DBS and DPS from non-stimulated and antigen stimulated whole blood samples. We applied a rigorous fit-for-purpose validation strategy [39], and evaluate the utility of the method for detection of immune responses towards *M.tuberculosis* and CMV.

## Materials and Methods

### QFT-TB Positive Individuals

We included samples from 18 individuals with a positive QFT-TB test from the Helsinki University Clinics. Thirteen were healthy individuals recently exposed to *M.tuberculosis*, four were patients with rheumatoid arthritis and candidates for anti-TNF treatment, and one was a suspect of but did not have active TB. The majority, 15, were from Finland, 2 were Somali and one was from Russia. Eight (44%) were females, median age was 57.5 (IQR 42–70 years).

### Healthy Controls

We included a total of 34 controls: 8 were volunteers among the staff members at the Clinical Research Centre, Hvidovre Hospital (control group 1); 26 were responding to a recruiting announcement (Control group 2). The control group 1 had a QFT-CMV test done and IP-10 was determined in DBS, DPS and plasma samples. The control group 2 had both QFT-TB and QFT-CMV tests done, and IP-10 in plasma and DPS samples as well as IFN-γ in plasma samples were analysed.

### QFT-TB and QFT-CMV Testing

For the antigen and mitogen stimulations we used the QFT-TB and QFT-CMV tubes (Cellestis/Qiagen, USA). The QFT-TB test comprise three heparinized vacutainer tubes; one TB antigen coated tube, one phytoheamaglutinin (PHA) coated tube, and one uncoated tube, The QFT-CMV test uses the same nil and PHA tube as the QFT-TB test in combination with a tube coated with CMV peptides. IFN-γ concentrations were measured using the QFT ELISA, the standard curve was extended to 16 IU/ml for better precision of high responses, as described previously [40]. QFT-TB and QFT-CMV results in plasma samples were analyzed according to manufacturers guidelines using manufacturers software (v2.61, www.cellestis.com).

### Ethical Considerations

The study and inclusion of the controls was approved by the Committees on Biomedical Research Ethics for the capital region of Denmark (H-3-2010-020). The ethical clearance to study samples from the tuberculosis suspects was from the Ethical committee from Helsinki University Central Hospital (Internal Diseases) (Drno. 301/E5/04). All participants provided written consent; the ethics committees approved this consent procedure.

### Monoclonal Antibodies and ELISA Development

**Figure 1:**
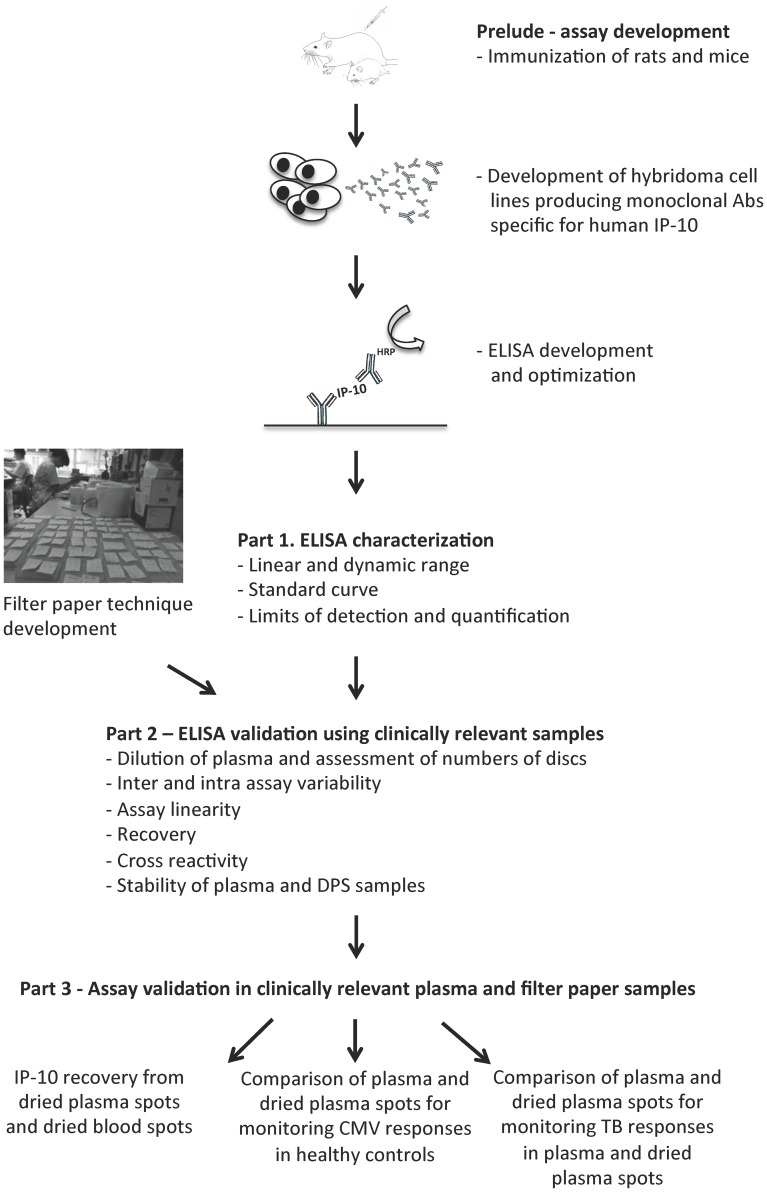
Overview of the development and validation process.

An overview of the individual experiments is presented in figure 1. We immunized 4 rats and 6 mice with recombinant human IP-10 (Peprotec, USA) and made 3 rat and 7 mouse hybridoma cell lines producing monoclonal antibodies (mAbs) specific for IP-10. Optimal capture and detection mAb concentration was determined using checkerboard titration for all potential mAb combinations. The strongest set of IP-10 binding mAbs was used for the IP-10 ELISA.

Maxisorb plates (Nunc, Denmark) were coated with murine capture mAbs (clone IM2) in carbonate buffer (pH 9.6) overnight at 4°C. The wells were saturated and proteins were stabilized with blocking buffer (0.1% BSA (Roche, DK), 5% trehalose (Sigma, DK) in PBS), buffer was poured out and plates were dried over night at 37°C. The plates were stable in gas-impermeable plastic bags with desiccant for at least 6 months (data not shown).

### Filter Paper Sample Preparation

Plasma or whole blood derived from QFT-TB or CMV tubes were spotted in 2×25 µL volume on Whatman 903 filter paper (Whatman, USA) per sample and allowed to air dry for 3–4 hours at ambient temperature in horizontal position over an open non-absorbent surface kept away from direct sunlight. Filter paper samples are stable and can be stored in gas-impermeable zip-lock plastic bags with a desiccant. On the day of analysis 2 filter paper discs of 5.5 mm were punched from the centre of the dried plasma or dried blood spots (DPS/DBS) using a standard office paper puncher (Impega, UK).

### Protocol for IP-10 Determination in Plasma DPS and DBS Samples

Plasma samples were diluted in 2 steps, first x10 in a non-absorbent 96-well mix plate (Nunc, Denmark) in dilution buffer (2% BSA, 0.1% Tween-20 in PBS), thereafter further dilution x3 in dilution buffer with HRP-conjungated detection mAb (clone IR1) and assayed in a final volume of 100 µL in the pre-coated ELISA plate.

For DPS and DBS samples, two filter paper discs were stacked horizontally in each ELISA plate well, and 100 µL assay buffer was added. Wells were checked for uneven stacking of discs and air bubbles between the discs are removed by gently pressing the discs together in the well using an empty pipette.

Plasma and filter paper samples were incubated for 2 hours at room temperature (23°C) and washed × 3 in wash buffer. TMB One (Trichem, DK) 100 µL was added, and the plates were revealed for 30 minutes before the colour reaction was stopped with 100 µL H_2_SO_4_ and absorbance was read at 450 nmsubtracted air blank 630 nm. A step-by-step guide for DPS/DBS sample preparation is presented in figure S1.

#### Statistics

We compared responses to antigens using non-parametric paired and non-paired tests (Wilcoxon and Mann-Whitney test). Standard curves were fitted with linear regression. Biomarker agreement was assessed using Bland-Altman plots and correlation with Spearman’s test. Cut off independent comparison of diagnostic potential was assessed with ROC curve analysis. A p-value <0.05 was considered significant. All analyses were done using GraphPad Prism v5.00 for Mac (GraphPad Software, USA).

## Results

### Part 1– ELISA Characteristics

We assessed the linear part of the ELISA by diluting recombinant IP-10 (Peprotec, USA) in 0.1% Casein (Sigma, USA) PBS buffer and plotting the ODs against the known concentrations. Linear range of the assay was 2.5–600 pg/ml (corresponding to 0.1–20 ng/ml in ×33 diluted plasma, figure S1). The dynamic range of the assay was 0−>10.000 pg/ml (figure S2). Standard curves were prepared by diluting recombinant IP-10 (Peprotec, USA), which were stored at −80°C until use. DPS and DBS measurements are presented as pg/2 DPS or DBS discs.

The ELISA had comparable precision if 2, 4 or 7 concentrations were used for the standard curve (table S1). Lower limit of detection (LOD) was 2.1 pg/ml determined as mean+3 s.d. ODs of the blank sample (n = 20, data not shown). Lower limit of quantification (LLOQ) was defined as the point at which the coefficient of variation for relevant patient samples is at least 15% [39]. The LLOQ was determined based on pooled nil, TB antigen and PHA mitogen samples from 18 patients with confirmed positive QFT-IT test (figure S3). Only 2 samples had a CV% >15% and these were both <4 pg/ml IP-10 (corresponding to <0.14 ng/ml after correcting for x33 dilution).

### Part 2–ELISA Validation

#### Dilution of plasma and number of filter paper discs

**Figure 2:**
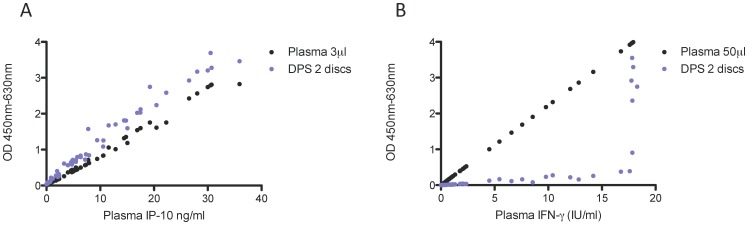
Magnitude of IP-10 (A) and IFN-γ (B) responses to antigen and mitogen challenge determined in plasma and DPS samples. Optical density reading from 18 Quantiferon-TB positive donors were compared. Nil, antigen and mitogen samples were pooled, rendering a total of 54 data-points per graph. OD readings from plasma and DPS samples were plotted against the IP-10 or IFN-g concentration determined in the plasma sample corrected for the dilution factor. The optimal working range of the assay is OD 0–3, a steadily increasing regression line ensures high precision.

We compared IP-10 and IFN-γresponses in nil, antigen and mitogen samples from 18 QFT-TB positive donors in plasma and DPS samples. IP-10 responses in plasma following stimulation with antigen and mitogen in the QFT-TB tubes were high and best fathomed within the linear part of the assay at x33 dilution ( = 3 µL plasma in 97 µL buffer per assay) or using two 5.5 mm DPS discs per measurement (figure 2A).

For comparison we also assessed levels of IFN-γ in plasma and DPS samples in the 18 QFT-TB positive donors (figure 2B). Despite the fact that all donors were QFT-TB positive, IFN-γ in DPS samples rendered very low OD readings, especially in the clinically relevant range around the cut off (0.35 IU/ml).

#### Comparison of plasma and DPS methods for IFN-γ and IP-10 detection

**Figure 3:**
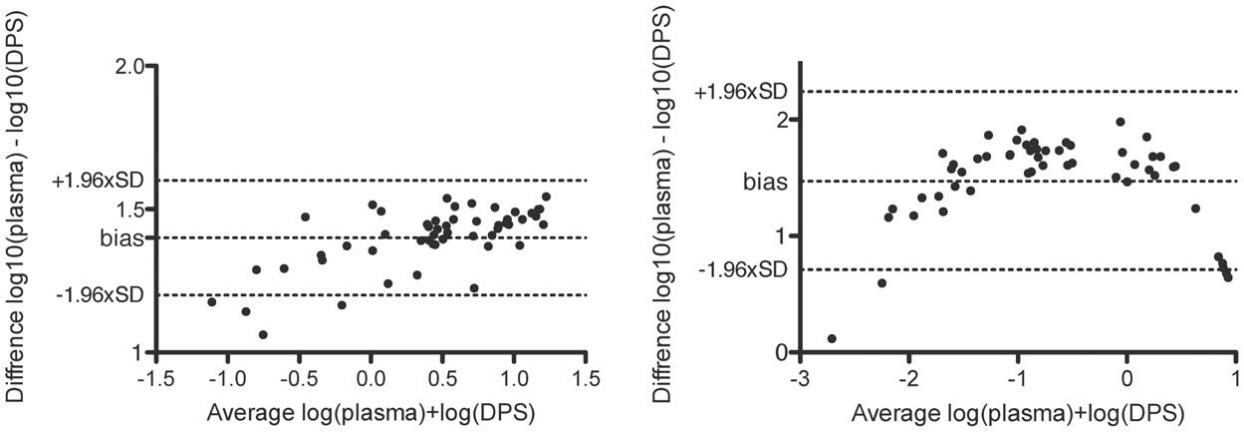
Agreement between IP-10 (A) and IFN-γ (B) detected in plasma and DPS samples. IP-10 and IFN-γ signals were detected in samples from 18 Quantiferon-TB positive donors; nil, antigen and mitogen samples were corrected for the dilution factor in plasma. Samples were pooled rendering a total of 54 data-points per graph. DPS concentrations were analyzed as ng/2 discs for IP-10 and IU/2 discs for IFN-γ signals (DPS) and log transformed to facilitate comparison between high (plasma) and low DPS. Each sample is represented on the graph as the average of the two measurements against the difference. The limits of agreement are specified as the average difference (bias) ±1.96 standard deviation of the difference.

The correlation between plasma and DPS was excellent for IP-10 (r^2^ = 0.97, p<0.001) and lower for IFN-γ (r^2^ = 0.56, p<0.001, data not shown). The agreement between plasma and DPS analysis was assessed in a Bland-Altman plot on the logged DPS and plasma concentrations (figure 3). The IP-10 plot suggests a good agreement between the two methods at average concentrations above 10^−0.5^ (0.3 ng/ml) ≈ 0.6 ng/ml in plasma and 24 pg/ml in DPS. In contrast the IFN-γ plot showed wide range in the limits of agreement and a high degree of heteroscedasticity at both low and high concentrations. As IFN-γ responses in DPS samples were very low and because the Bland-Altman demonstrated systematic bias - also in the clinical relevant area around the cut off - we concluded that the filter paper method was unreliable for the assessment of IFN-γ. We did not pursue IFN-γ further with the filter paper technique.

#### ELISA validation experiments

The between- and within- and total-run imprecision was assessed for plasma, DPS and DBS samples as described by Krouwer et al [41]. The assay imprecision was acceptable for all three types of sample, demonstrating a within-run imprecision below 10 CV%, a between-run imprecision below 8 CV% and a total assay imprecision below 11% (table S2 and S3). The assay had a high degree of linearity with recovery of 85–125% in plasma samples examined at dilutions from × 4 to × 64 (table S4). The recovery of recombinant IP-10 added to plasma was high 95–110% (table S5) and we observed no cross-reactivity with any of the 25 cytokines and chemokines examined table S6). We further investigated the stability of IP-10 in plasma, DPS and DBS. IP-10 was stable in DPS samples for at least 4 weeks at up to 37°C and at up to 50°C for up to 2 weeks (table S7). Plasma concentrations of IP-10 were unaffected by 6 weeks storage at +5°C (table S8), 2 weeks storage at 23°C (Table S9) and 10 freeze thaw cycles (table S10).

#### Difference in magnitude of response between plasma and DPS samples

We compared the difference in magnitude of CMV and *M.tuberculosis* specific IP-10 and IFN-γ release head-to-head. Only samples from QFT-TB (n = 14) and QFT CMV (n = 13) positive donors that were within the linear range of both assays were included. IP-10 was induced in median 99-fold higher levels (range 22–470-fold, p<0001).

### Part 3 - Assay Validation in Clinically Relevant Plasma and Filter Paper Samples

#### IP-10 recovery from DPS and DBS

QFT-CMV responses from 8 healthy individuals (control group 1) were used to compare IP-10 in DBS versus DPS. Three donors were IP-10-CMV non-responders with a median IP-10 response to CMV antigens of 0 pg/2 DPS discs (range 0–0 pg/2 DPS discs), 5 were IP-10-CMV responders with a median of 560 pg/2 DPS discs, (range 79–1169 pg/2 DPS discs)). All donors responded to PHA (median 475 pg/2 DPS discs (range 83–924 pg/2 DPS discs)).

**Figure 4:**
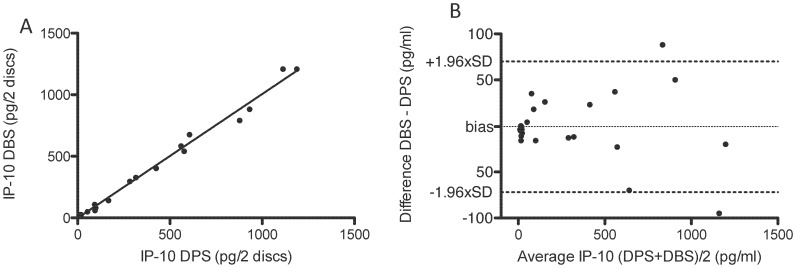
IP-10 responses in DBS compared to DPS. QFT-CMV test stimulated blood from 8 healthy individuals, 5 CMV responders, were used to compare IP-10 signals in DBS and DPS samples. Nil antigen and mitogen samples pooled (n = 24). Figure 4A. Correlation analysis, (p<0.0001, Spearman); Figure 4B Bland-Altman plot of the same data. Each sample is represented on the graph as the average of the two measurements against the difference. The limits of agreement are specified as the average difference (bias, −0.8 pg/ml) ±1.96 standard deviation of the difference, (71 pg/ml).

There was high correlation (r^2^ = 0.99) and identical recovery of IP-10 signal from DPS and DBS samples (regression slope = 1.01, figure 4A). The mean difference was 0.8 pg/ml (SD 36.2 pg/ml) with a constant scatter of the differences around mean at concentrations below 500 pg/ml indicating high agreement between the two methods (figure 4B). The regression analysis suggested that the signal intensity from 2 filter paper discs corresponds to a volume of blood or plasma of approximately 3.6 µL per 100 µL ELISA well volume (3 µl/0.83). Two filter paper discs (with a diameter of 5.5 mm) have the area of 47.6 mm^2^, i.e. 1 µL plasma generates a signal corresponding to 13.2 mm^2^ of DPS or DBS if analysed in a 100 µL ELISA well (47.6 mm^2^/3.6 µL).

#### Comparison of the plasma and DPS method for monitoring CMV responses in healthy controls

**Figure 5:**
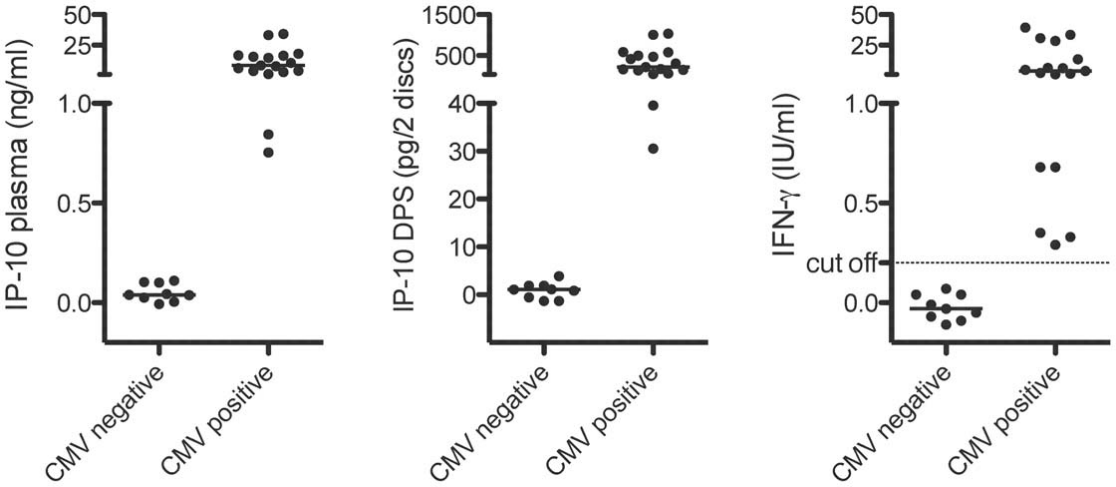
IP-10 responses in plasma and DPS. QFT-CMV responses from 26 healthy individuals (Control group 2) were divided into CMV-positive (n = 17) and CMV-negative (n = 9) based on the QFT-CMV cut off for IFN-γ suggested by the manufacturer (0.2 IU/ml).

**Figure 6:**
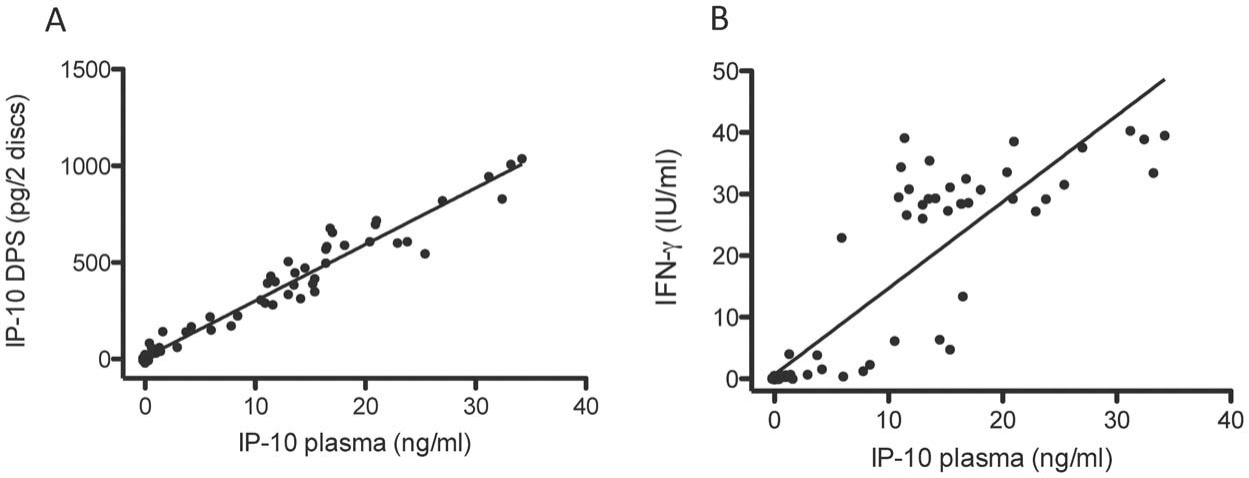
Correlation between IP-10 detected in plasma and DPS (A), and between IP-10 detected in plasma and INF-γ detected in plasma (B). A total of 25 healthy donors were compared. Nil, CMV-antigen and mitogen samples were pooled, rendering a total of 75 data-points per graph (Spearman).

QFT-CMV responses from 26 healthy individuals (Control group 2) were compared (figure 5). We divided the individuals into CMV-positive (n = 17) and CMV-negative (n = 9) based on the QFT-CMV cut off for IFN-γ suggested by the manufacturer (0.2 IU/ml). The division of the controls in the two groups was reproduced by the IP-10 assays. This small set of data suggests a cut-off for CMV positivity in the range 0.11–0.75 ng/ml for IP-10 in plasma and 4–30 pg for 2 DPS discs. Figure 6 shows IP-10 plasma levels plotted against DPS levels from pooled nil, QFT-CMV and PHA samples. We found a very high correlation also in this group of controls (r^2^ = 0.97, p<0.0001); the correlation between plasma IP-10 and plasma IFN-γ was lower (r^2^ = 0.81, p<0.0001).

#### Comparison of the plasma and DPS method for measuring *M. tuberculosis* specific immune responses in QFT-TB positive patients and controls

We assessed the diagnostic potential of the IP-10 ELISA in plasma and DPS samples in comparison with IFN-γ in samples from 18 QFT-TB positive patients and 26 controls (control group 2). All controls tested QFT-TB negative. In TB patients the antigen and mitogen responses were significantly higher than non-stimulated values (p<0.0001 for all), and non-stimulated values were significantly higher among QFT-TB positive compared to controls (p<0.002) (table 1). The diagnostic potential of IP-10 in DPS and plasma samples was analysed in ROC curve analysis; the AUC was 1.00 (95% CI 1.00–1.00) for both methods.

**Table 1:** 

		IP-10		QFT-TB
		Plasma (ng/ml)	DPS (pg/2 discs)	Plasma (IU/ml)
QFT-TB positive	Nil	0.8 (0.2–2.3)	38 (16–94)	0.1 (0.0–0.2)
median (IQR)	Antigen-Nil	9.7 (6.6–15.0)	311 (233–572)	2.0 (0.9–7.8)
	Mitogen-Nil	15.4 (5.4–21.1)	547 (223–805)	8.7 (1.3–16.9)
Healthy controls	Nil	0.0 (0.0–0.2)	8 (4–15)	0.0 (0.0–0.0)
median (IQR)	Antigen-Nil	0.0 (0.0–0.1)	1 (0–4)	0.0 (0.0–0.0)
	Mitogen-Nil	15.3 (12.1–21.0)	475 (358–643)	29.4 (28.3–34.2)

IP-10 plasma and DPS responses in 18 QFT-TB positive and 25 healthy controls compared to IFN-γ levels in plasma.

## Discussion

In this study we have introduced a novel ELISA based method for quantification IP-10 in blood and plasma samples: dried plasma (DPS) and dried blood spots (DBS). The ELISA was rigorously validated and demonstrated acceptable performance within the clinically relevant range of IP-10 concentrations. We demonstrated that IP-10 is stable on filter paper with prolonged storage at high temperatures, and showed that IP-10 determined in DPS/DBS and plasma correlated very well within the whole range of the assay. Finally, we applied the method to clinical samples and demonstrated that the method is reliable for determining immune responses to *M. tuberculosis* and CMV antigens challenge, and has a diagnostic potential.

IP-10 is a promising readout marker for infection with *M.tuberculosis* vis-a-vis IFN-γ in the IGRAs [2], and CMV provides a good model to study chronic or latent infections. IP-10 mRNA induction has previously been used as a readout marker for CMV infection [42], and the IFN-γ based QFT-CMV assay was recently introduced as a monitoring tool for CMV infection in immunosuppressed individuals [43]. The obtained results suggest that IP-10 and the filter paper-based assays are valid for CMV detection and have comparable performance to the QFT-CMV assay, and consolidate IP-10 as a marker for infection with *M.tuberculosis*.

### Cytokines in Filter Paper, History and Discussion of Results

We have explored the use of filter paper as a simple method of storing and transporting samples for later analysis. Skogstrand and co-workers were the first to detect cytokine and chemokine sin DBS using an in-house multiplex assay applied to DBS discs [37], [44], [45]. Very recently the group extended their investigations to DBS samples from QFT-TB stimulated blood [46]. The study found higher levels of IFN-γ, IL-2, GM-CSF, IL-5 and IL-1β in QFT-TB positive samples compared to QFT-TB negative samples (p<0.05), but did not confirm previous reports suggesting a diagnostic potential for MCP-1, TNF-α, IL-8 and IL-10 [47]–[53]. The multiplex assay detected low IFN-γ levels from the DBS samples and the majority of measurements from both patients and control samples fell below the lower limit of quantification reported for the assay (defined as <20 CV%) [44]. These results are in line with our findings of very low OD signals using the QFT ELISA on DPS samples.

The reason for the poor performance of IFN-γ compared to IP-10 is likely explained by the lower volume of plasma available in the DPS samples compared to a standard QFT ELISA. The multiplex study [46] did not present IFN-γ plasma levels measured with the QFT ELISA and it is therefore not possible to assess if the indication of diagnostic potential is due to high IFN-γ levels in the QFT-TB positive samples in the study. Results from the multiplex DBS assay suggest an unexpected poor stability for some cytokines in DBS samples. IFN-γ levels increases 2-fold if stored at 30 days at room temperature and decreases 1.4 fold if stored at +4°C for 30 days [45]. This instability of IFN-γ in DBS samples could influence the findings and limits the potential of IFN-γ as a readout marker in an assay with a fixed cut off. Taken together, IFN-γ and other biomarkers expressed in low levels are poor candidates for this kind of assay.

### Biomarker Validation

We aimed to fathom typical response levels from non-stimulated and stimulated samples. IP-10 is a biomarker expressed at very high levels in response to stimulation, wherefore we had to dilute plasma extensively to retain the IP-10 concentrations induced with stimulation within the assays range and the reference control level low but above the LLOQ.

Dilution of plasma samples for IP-10 detection is essential. We recently conducted a review of the 22 published studies exploring the use of IP-10 as a diagnostic marker for TB in CMI assays and found little consensus of which assays - and importantly - which dilution is optimal for IP-10 detection [2]. Clearly, our extensive dilution of plasma samples is the most extreme yet published for IP-10, but compared to the signal intensity generated by 50 µL plasma in the IFN-γ assay, it appears conservative.

Several reports suggest that IP-10 in non-stimulated plasma samples can be used to monitor treatment efficacy of tuberculosis [10], [11], [54], [55]; and also malaria [12]. Further, IP-10 assays have also shown promise for liver fibrosis monitoring in patients with chronic hepatitis C viral infection (HCV) [7], [56]. We have validated the filter paper assay in patients with chronic HCV infection and advanced liver fibrosis compared to no fibrosis and demonstrated comparable performance to plasma (Ruhwald et al, unpublished).

### Signal Intensity in DBS Versus DPS

Analysis from DPS and DBS samples produced equal responses, despite the very different composition and viscosity of whole blood compared to plasma. DBS samples have operational advantages over DPS as DBS samples can be obtained from a finger prick blood and an IP-10 release assay can be performed without centrifugation.

### Limitations

Most of the validation experiments were based on small numbers. This is acceptable at the exploratory method validation step, but a larger later-stage validation of the assay in clinical relevant settings is important [39]. We only present data on Whatman903 filter paper. Thicker filter paper retain higher sample volume, and could likely enable the desired IP-10 signal intensity readings from a single disc, and potentially improve the assay for IFN-γ. Whether thicker paper can bring the IFN-γ signals into the range of reliable detection is unlikely as one would need a plasma spot area of 660 mm2 Whatman 903 or the equivalent of 28 5.5 mm diameter DPS discs to generate signal comparable to 50 µL plasma. We have generated data using other types of filter paper including Whatman 3 MM which has proven reliable for other settings [36]. We did not extend the full validation protocol to the DBS samples, but performed the key stability and reproducibility assessments and obtained the same reliable results as for DBS (data not shown). Based on these experiments and the notion that the DBS and DPS methods both are considered very reliable [29], [57], [58], we felt confident that the two methods were comparable.

### Perspectives

Further studies are needed to consolidate these findings, and results from our group demonstrate that the IP-10 filter paper method is valid for the diagnosis of infection with *M.tuberculosis* in a CMI assay (Aabye et al, unpublished) and for monitoring of liver fibrosis in patients with chronic infection with hepatitis C (Ruhwald et al unpublished). Studies have suggested that changes in blood levels of IP-10 (non-stimulated samples) can be used as a marker for treatment effect in patients with TB disease, wherefore the filter paper method could be employed as a simple and quick monitoring tool based on finger prick blood. This filter paper based approach can further be combined with DBS based therapeutic drug monitoring as recently suggested by Vu and colleges [59] e.g. applicable for routine use in resource limited settings. Finally, as IP-10 is detectable in very small volumes of samples, IP-10 release assays can be miniaturized as recently suggested by Kasprowicz and co-workers using a qPCR assay for IP-10 detection in as little as 50 µL antigen stimulated whole blood [42].

### Conclusion

We developed and validated a novel sensitive and robust assay for detection of IP-10 and demonstrated its use in plasma, DPS and DBS samples from antigen stimulated and non-stimulated whole blood.

## Supporting Information

Figure S1 **Preparation of filter paper for QFT supernatants.** Draw 3×2 dots for nil (black), antigen (red) and mitogen (blue/purple) application. **Sample preparation for frozen samples (skip if using fresh plasma)** 1) Defrost QFT samples. 2) Vortex 20 sec. per sample. 3) Centrifuge to pellet debris (e.g. 2000 RPM for 2 min) **A. Adding sample** 1) Place the filter paper on a open non-absorbent surface and kept away from direct sunlight (e.g. an empty pipette tip box or drying rack). 2) Add 25 µL plasma on each dot – i.e. 2×25 µL from each QFT tube. 3) Allow to dry for 3–4 hours **B. Sample transport and storage** 1) Place filter papers in sealed zip-bags with desiccant and an optional humidity indicator card. 2) For storage place the zip-bag(s) in another zip-bag and store cold (preferably at −20C). 3) Ship with normal post at ambient temperature **C–E. Assay** 1) Punch 2 5.5 mm disc. 2) Stack two discs in each ELISA plate well. 3) Add 100 µL assay buffer. 4) Check for uneven stacking of discs and air bubbles, remove bubbles by gently pressing the discs together. 5) Incubate 2 hours at ambient temperature (in the dark). 6) Wash plate ×3, be sure all discs are knocked out. 7) Add TMB substrate. 8) Stop reaction and read plate.
(TIFF)

Figure S2 **Typical standard curve (A) and dynamic range (B) of the IP-10 ELISA.** Typical standard curve (A) and dynamic range (B) of the IP-10 ELISA A. Recombinant IP-10 (Peprotec, USA) was prepared in 7 concentrations: 1.9 pg/ml, 9.4 pg/ml, 38 pg/ml, 94 pg/ml, 189 pg/ml, 377 pg/ml and 566 pg/ml and analyzed using the IP-10 ELISA, presented is a typical example of a standard curve. B. Recombinant IP-10 was serially diluted (from 13 ng/ml) and analyzed using the IP-10 ELISA. The assay was stopped when the highest concentration of IP-10 was >3 OD to prevent artificial quenching of the signal.
(TIFF)

Figure S3 **Lower Limit of Quantification (LLOQ).** LLOQ can be defined as the point at which the coefficient of variation for relevant patient samples is at least 15% [38]. The LLOQ was determined based on pooled nil, TB antigen and PHA mitogen samples from 18 patients with confirmed positive QFT-IT test. Only 2 samples had a CV% >15% and these were both <4 pg/ml IP-10 (corresponding to <0.14 ng/ml after correcting for x33 dilution).
(TIFF)

Table S1 **Linearity of the standard curve.** We performed 48 consecutive IP-10 assays including aliquots of the same plasma sample and aliquots of the same 7-point standard curve. We prepared standard curves by diluting recombinant IP-10 (Peprotec, USA) in the following 7 concentrations: 566 pg/ml, 377 pg/ml, 189 pg/ml, 94 pg/ml, 38 pg/ml, 9.4 pg/ml and 1.9 pg/ml, for standard point 1 to 7 respectively). We analyzed the impact of the total number of points in the standard curve and the importance of the individual points by reducing the number of points from seven to three, two or one concentration. Standard curves with a reduced number of points were fully comparable to the 7-point standard curve regarding slope and precision of the IP-10 concentration in the plasma sample.
(DOCX)

Table S2 **Estimates of assay imprecision.** Within-run, Between-run and Total imprecision of the assays was determined using 4 representative samples. DBS, DPS and plasma samples (at x33 dilution) were prepared and analyzed in 5-replicates in 5 independent assays performed by the same operator using the same equipment. The experiments describe the random error caused by operator, assay, instrument and day variation. Sample 1 was below the LLOQ of the assay and has as expected higher degree of imprecision. For the 3 samples within the range of the assay; both within-run, between-run and total assay imprecision was below our acceptance criteria: <10%, 15% and 15%, respectively.
(DOCX)

Table S3 **Within- and between-run imprecision.** A. Dried blood spot (DBS) samples (pg/2 discs). B. Dried plasma spot (DBS) samples (pg/2 discs). C. Plasma samples (ng/ml).
(DOCX)

Table S4 **Assay Linearity.** Linearity was determined in 4 plasma samples serially diluted throughout the assay range. The % recovery was calculated as observed vs. expected concentration. Average linearity was within our acceptance range of 70–130% between x4 and x64 dilution.
(DOCX)

Table S5 **Spike-recovery of IP-10 in plasma samples.** Spike recovery was performed by spiking recombinant IP-10 (Peprotec, USA) into ×10 and ×3 diluted plasma samples without detectable IP-10. The average % recovery was calculated as the proportion of spiked standard in the sample (observed) to that of the control spike (expected).
(DOCX)

Table S6 **Cross reactivity with similar analytes.** We observed no interference with the following analytes at the concentration in parenthesis: TNF-a (1.2 ng/ml), IL-1b (3.19 ng/ml), IL-2 (1.7 ng/ml), IL-4 (16.18 ng/ml), IL-5 (4.14 ng/ml), IL-6 (3.71 ng/ml), IL-8 (11.17 ng/ml), IL-10 (9.2 ng/ml), IL-3 (1.48 ng/ml), IL-7 (7.64 ng/ml), IL-1a (20.5 ng/ml), IL-12p40/p70 (7.14 ng/ml), IL-13 (6.35 ng/ml), IL-15 (11.6 ng/ml), IL-17 (7.8 ng/ml), IFN-a (5.3 ng/ml), IFN-γ (5.2 ng/ml), GM-CSF (6.5 ng/ml), MCP-1 (10.2 ng/ml), MIP1α (12.5 ng/ml), MIP1β (5.8 ng/ml), Eotaxin (1.7 ng/ml), RANTES (5.2 ng/ml), MIG (1.3 ng/ml), Recombinant protein (all from Invitrogen, USA) were diluted in assay buffer and analysed in 2 pools of 10 (first 10 on list) and 25 using the IP-10 ELISA.
(DOCX)

Table S7 **Stability of IP-10 in dried plasma spots.** Stability studies determine the longest time IP-10 in a DPS sample can be stored before it deteriorates and produce inaccurate results. Stability was determined by leaving DPS samples at 5°C, 23°C, 37°C and 50°C for 0–4 weeks as listed in the table 7 A–D. Recovery was within our acceptance range of 70–130%, indicating that DPS samples can be safely stored at +5°C–37°C for at least 4 weeks without significant loss in recovery, and at 50°C for up to 2 weeks. Similar results were obtained for whole blood samples (data not shown).
(DOCX)

Table S8 **Stability of IP-10 in plasma stored at +5°C for up to 6 weeks.** The stability of IP-10 in plasma was determined by leaving samples at 5°C for the weeks listed in the table. Samples were within our acceptance range of 70–130%, indicating that samples can be stored at +5°C for at least 6 weeks with no loss of signal.
(DOCX)

Table S9 **Stability of IP-10 in plasma with storage at room temperature +23°C.** The stability of IP-10 in a plasma sample stored at room temperature was assessed. %Recovery is calculated by comparing the value of the treated sample to the reference (0 days) sample ×100. Samples were within our acceptance range of 70–130%, indicating samples can be stored at room temperature for prolonged periods without significant loss in recovery.
(DOCX)

Table S10 **Stability of IP-10 in plasma with repeated freeze thaw cycles.** The ability of IP-10 in plasma to tolerate freeze-thaw cycles was assessed by freezing and thawing up to 10 times. Samples were thawed at room temperature and left for minimum 2 hours before refreezing. There was at least 24 hours between each freeze-thaw cycle. %Recovery is calculated by comparing the value of the treated sample to the freshly thawed control sample x100. Samples were within our acceptance range of 70–130%, indicating samples can undergo at least 10 freeze-thaw cycles without major loss in recovery.
(DOCX)
