# Supplementary figures and images for: A Simple Method to Quantitate IP-10 in Dried Blood and Plasma Spots
Source: PLoS One. 2012 Jun 27;7(6):e39228. doi: 10.1371/journal.pone.0039228 (PMC3384664; doi:10.1371/journal.pone.0039228)

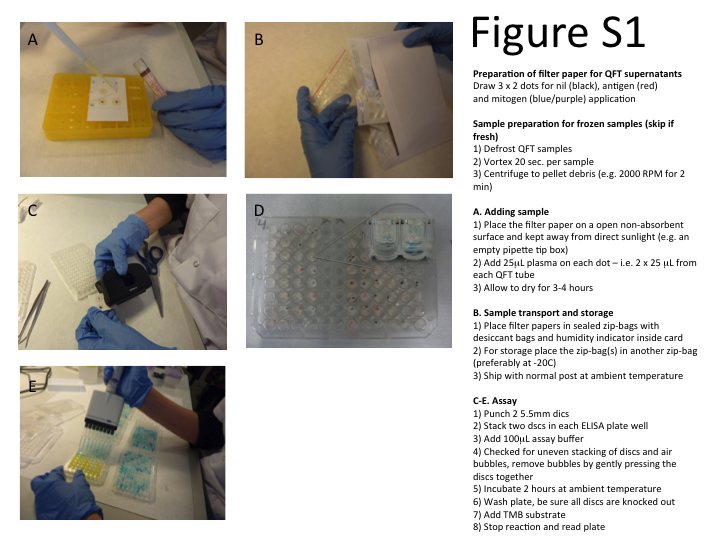

Supplement: Figure S1 — Preparation of filter paper for QFT supernatants. Draw 3×2 dots for nil (black), antigen (red) and mitogen (blue/purple) application. Sample preparation for frozen samples (skip if using fresh plasma) 1) Defrost QFT samples. 2) Vortex 20 sec. per sample. 3) Centrifuge to pellet debris (e.g. 2000 RPM for 2 min) A. Adding sample 1) Place the filter paper on a open non-absorbent surface and kept away from direct sunlight (e.g. an empty pipette tip box or drying rack). 2) Add 25 µL plasma on each dot – i.e. 2×25 µL from each QFT tube. 3) Allow to dry for 3–4 hours B. Sample transport and storage 1) Place filter papers in sealed zip-bags with desiccant and an optional humidity indicator card. 2) For storage place the zip-bag(s) in another zip-bag and store cold (preferably at −20C). 3) Ship with normal post at ambient temperature C–E. Assay 1) Punch 2 5.5 mm disc. 2) Stack two discs in each ELISA plate well. 3) Add 100 µL assay buffer. 4) Check for uneven stacking of discs and air bubbles, remove bubbles by gently pressing the discs together. 5) Incubate 2 hours at ambient temperature (in the dark). 6) Wash plate ×3, be sure all discs are knocked out. 7) Add TMB substrate. 8) Stop reaction and read plate. (TIFF) [file pone.0039228.s001.tiff]

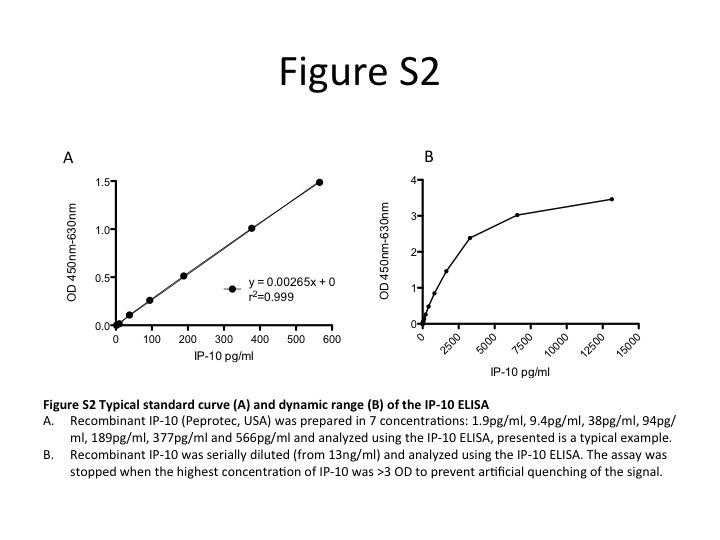

Supplement: Figure S2 — Typical standard curve (A) and dynamic range (B) of the IP-10 ELISA. Typical standard curve (A) and dynamic range (B) of the IP-10 ELISA A. Recombinant IP-10 (Peprotec, USA) was prepared in 7 concentrations: 1.9 pg/ml, 9.4 pg/ml, 38 pg/ml, 94 pg/ml, 189 pg/ml, 377 pg/ml and 566 pg/ml and analyzed using the IP-10 ELISA, presented is a typical example of a standard curve. B. Recombinant IP-10 was serially diluted (from 13 ng/ml) and analyzed using the IP-10 ELISA. The assay was stopped when the highest concentration of IP-10 was >3 OD to prevent artificial quenching of the signal. (TIFF) [file pone.0039228.s002.tiff]

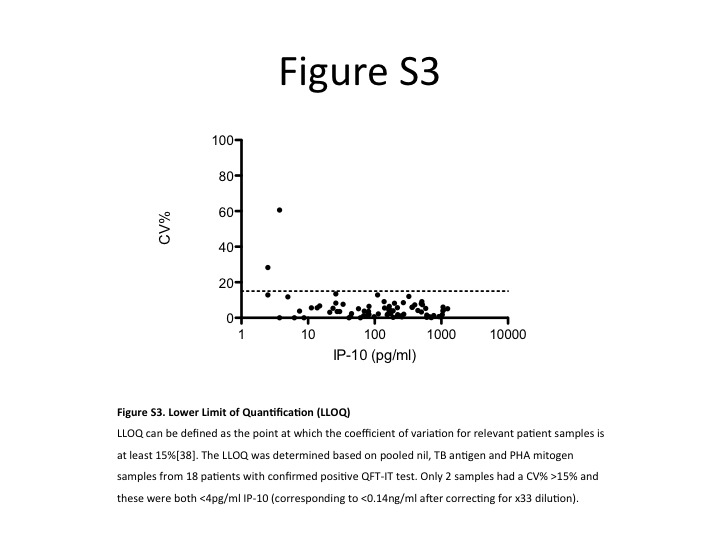

Supplement: Figure S3 — Lower Limit of Quantification (LLOQ). LLOQ can be defined as the point at which the coefficient of variation for relevant patient samples is at least 15% [38]. The LLOQ was determined based on pooled nil, TB antigen and PHA mitogen samples from 18 patients with confirmed positive QFT-IT test. Only 2 samples had a CV% >15% and these were both <4 pg/ml IP-10 (corresponding to <0.14 ng/ml after correcting for x33 dilution). (TIFF) [file pone.0039228.s003.tiff]
