# Supplementary material for: A Simple Method to Quantitate IP-10 in Dried Blood and Plasma Spots
Source: PLoS One. 2012 Jun 27;7(6):e39228. doi: 10.1371/journal.pone.0039228 (PMC3384664; doi:10.1371/journal.pone.0039228)
Supplement: Table S1 — Linearity of the standard curve. We performed 48 consecutive IP-10 assays including aliquots of the same plasma sample and aliquots of the same 7-point standard curve. We prepared standard curves by diluting recombinant IP-10 (Peprotec, USA) in the following 7 concentrations: 566 pg/ml, 377 pg/ml, 189 pg/ml, 94 pg/ml, 38 pg/ml, 9.4 pg/ml and 1.9 pg/ml, for standard point 1 to 7 respectively). We analyzed the impact of the total number of points in the standard curve and the importance of the individual points by reducing the number of points from seven to three, two or one concentration. Standard curves with a reduced number of points were fully comparable to the 7-point standard curve regarding slope and precision of the IP-10 concentration in the plasma sample. (DOCX) [file pone.0039228.s004.docx]

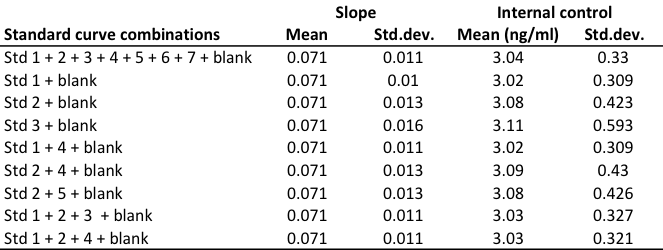


**Table S1 Linearity of the standard curve**

We performed 48 consecutive IP-10 assays including aliquots of the same plasma sample and aliquots of the same 7-point standard curve. We prepared standard curves by diluting recombinant IP-10 (Peprotec, USA) in the following 7 concentrations: 566pg/ml, 377pg/ml, 189pg/ml, 94pg/ml, 38pg/ml, 9.4pg/ml and 1.9pg/ml, for standard point 1 to 7 respectively). We analyzed the impact of the total number of points in the standard curve and the importance of the individual points by reducing the number of points from seven to three, two or one concentration. Standard curves with a reduced number of points were fully comparable to the 7-point standard curve regarding slope and precision of the IP-10 concentration in the plasma sample.
