# Supplementary material for: A Simple Method to Quantitate IP-10 in Dried Blood and Plasma Spots
Source: PLoS One. 2012 Jun 27;7(6):e39228. doi: 10.1371/journal.pone.0039228 (PMC3384664; doi:10.1371/journal.pone.0039228)
Supplement: Table S2 — Estimates of assay imprecision. Within-run, Between-run and Total imprecision of the assays was determined using 4 representative samples. DBS, DPS and plasma samples (at x33 dilution) were prepared and analyzed in 5-replicates in 5 independent assays performed by the same operator using the same equipment. The experiments describe the random error caused by operator, assay, instrument and day variation. Sample 1 was below the LLOQ of the assay and has as expected higher degree of imprecision. For the 3 samples within the range of the assay; both within-run, between-run and total assay imprecision was below our acceptance criteria: <10%, 15% and 15%, respectively. (DOCX) [file pone.0039228.s005.docx]

**Table S2A-C. Estimates of assay imprecision**

**Table 2A. Dried Blood Spots (DBS)**

**Table 2B. Dried Plasma Spots (DPS)**

**Table 2C. Plasma**

**Table S2A-C. Estimates of assay imprecision**

Within-run, Between-run and Total imprecision of the assays was determined using 4 representative samples. DBS, DPS and plasma samples (at x33 dilution) were prepared and analyzed in 5-replicates in 5 independent assays performed by the same operator using the same equipment. The experiments describe the random error caused by operator, assay, instrument and day variation.

Sample 1 was below the LLOQ of the assay and has as expected higher degree of imprecision. For the 3 samples within the range of the assay; both within-run, between-run and total assay imprecision was below our acceptance criteria: <10%, 15% and 15%, respectively.
