# Supplementary material for: A Simple Method to Quantitate IP-10 in Dried Blood and Plasma Spots
Source: PLoS One. 2012 Jun 27;7(6):e39228. doi: 10.1371/journal.pone.0039228 (PMC3384664; doi:10.1371/journal.pone.0039228)
Supplement: Table S3 — Within- and between-run imprecision. A. Dried blood spot (DBS) samples (pg/2 discs). B. Dried plasma spot (DBS) samples (pg/2 discs). C. Plasma samples (ng/ml). (DOCX) [file pone.0039228.s006.docx]

**Table 3A. Raw values - Dried blood spot (DBS) samples (pg/2 discs)**

**Table 3B. Raw values - Dried plasma spot (DBS) samples (pg/2 discs)**

**Table 3C. Raw values - Plasma samples (ng/ml)**
