# Supplementary material for: A Simple Method to Quantitate IP-10 in Dried Blood and Plasma Spots
Source: PLoS One. 2012 Jun 27;7(6):e39228. doi: 10.1371/journal.pone.0039228 (PMC3384664; doi:10.1371/journal.pone.0039228)
Supplement: Table S4 — Assay Linearity. Linearity was determined in 4 plasma samples serially diluted throughout the assay range. The % recovery was calculated as observed vs. expected concentration. Average linearity was within our acceptance range of 70–130% between x4 and x64 dilution. (DOCX) [file pone.0039228.s007.docx]

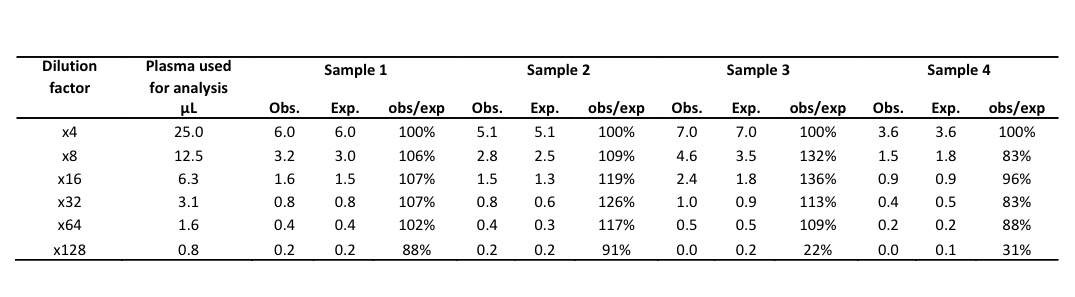


**Table S4. Assay Linearity**

Linearity was determined in 4 plasma samples serially diluted throughout the assayrange. The % recovery was calculated as observed vs. expected concentration. Average linearity was within our acceptance range of 70-130% between x4 and x64 dilution
