# Supplementary material for: A Simple Method to Quantitate IP-10 in Dried Blood and Plasma Spots
Source: PLoS One. 2012 Jun 27;7(6):e39228. doi: 10.1371/journal.pone.0039228 (PMC3384664; doi:10.1371/journal.pone.0039228)
Supplement: Table S5 — Spike-recovery of IP-10 in plasma samples. Spike recovery was performed by spiking recombinant IP-10 (Peprotec, USA) into ×10 and ×3 diluted plasma samples without detectable IP-10. The average % recovery was calculated as the proportion of spiked standard in the sample (observed) to that of the control spike (expected). (DOCX) [file pone.0039228.s008.docx]

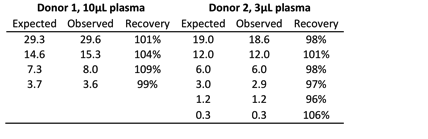


**Table S5. Spike-recovery of IP-10 in plasma samples**

Spike recovery was performed by spiking recombinant IP-10 (Peprotec, USA) into x10 and x3 diluted plasma samples without detectable IP-10. The average % recovery was calculated as the proportion of spiked standard in the sample (observed) to that of the control spike (expected).
