# Supplementary material for: A Simple Method to Quantitate IP-10 in Dried Blood and Plasma Spots
Source: PLoS One. 2012 Jun 27;7(6):e39228. doi: 10.1371/journal.pone.0039228 (PMC3384664; doi:10.1371/journal.pone.0039228)
Supplement: Table S6 — Cross reactivity with similar analytes. We observed no interference with the following analytes at the concentration in parenthesis: TNF-a (1.2 ng/ml), IL-1b (3.19 ng/ml), IL-2 (1.7 ng/ml), IL-4 (16.18 ng/ml), IL-5 (4.14 ng/ml), IL-6 (3.71 ng/ml), IL-8 (11.17 ng/ml), IL-10 (9.2 ng/ml), IL-3 (1.48 ng/ml), IL-7 (7.64 ng/ml), IL-1a (20.5 ng/ml), IL-12p40/p70 (7.14 ng/ml), IL-13 (6.35 ng/ml), IL-15 (11.6 ng/ml), IL-17 (7.8 ng/ml), IFN-a (5.3 ng/ml), IFN-γ (5.2 ng/ml), GM-CSF (6.5 ng/ml), MCP-1 (10.2 ng/ml), MIP1α (12.5 ng/ml), MIP1β (5.8 ng/ml), Eotaxin (1.7 ng/ml), RANTES (5.2 ng/ml), MIG (1.3 ng/ml), Recombinant protein (all from Invitrogen, USA) were diluted in assay buffer and analysed in 2 pools of 10 (first 10 on list) and 25 using the IP-10 ELISA. (DOCX) [file pone.0039228.s009.docx]

**Table S6. Cross reactivity with similar analytes**

We observed no interference with the following analytes at the concentration in parenthesis:

TNF-a (1.2ng/ml)

IL-1b (3.19ng/ml)

IL-2 (1.7ng/ml)

IL-4 (16.18ng/ml)

IL-5 (4.14ng/ml)

IL-6 (3.71ng/ml)

IL-8 (11.17ng/ml)

IL-10 (9.2ng/ml)

IL-3 (1.48ng/ml)

IL-7 (7.64ng/ml)

IL-1a (20.5ng/ml)

IL-12p40/p70 (7.14ng/ml)

IL-13 (6.35ng/ml)

IL-15 (11.6ng/ml)

IL-17 (7.8ng/ml)

IFN-a (5.3ng/ml)

IFN-γ (5.2ng/ml)

GM-CSF (6.5ng/ml)

MCP-1 (10.2ng/ml)

MIP1α (12.5ng/ml)

MIP1β (5.8ng/ml)

Eotaxin (1.7ng/ml)

RANTES (5.2ng/ml)

MIG (1.3ng/ml)

Recombinant protein (all from Invitrogen, USA) were diluted in assay buffer and analysed in 2 pools of 10 (first 10 on list) and 25 using the IP-10 ELISA.
