# Supplementary material for: A Simple Method to Quantitate IP-10 in Dried Blood and Plasma Spots
Source: PLoS One. 2012 Jun 27;7(6):e39228. doi: 10.1371/journal.pone.0039228 (PMC3384664; doi:10.1371/journal.pone.0039228)
Supplement: Table S7 — Stability of IP-10 in dried plasma spots. Stability studies determine the longest time IP-10 in a DPS sample can be stored before it deteriorates and produce inaccurate results. Stability was determined by leaving DPS samples at 5°C, 23°C, 37°C and 50°C for 0–4 weeks as listed in the table 7 A–D. Recovery was within our acceptance range of 70–130%, indicating that DPS samples can be safely stored at +5°C–37°C for at least 4 weeks without significant loss in recovery, and at 50°C for up to 2 weeks. Similar results were obtained for whole blood samples (data not shown). (DOCX) [file pone.0039228.s010.docx]

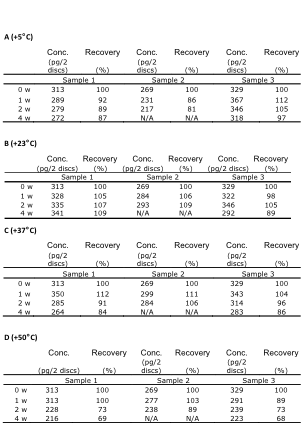


**Table S7 – stability of IP-10 in dried plasma spots**

Stability studies determine the longest time IP-10 in a DPS sample can be stored before it deteriorates and produce inaccurate results. Stability was determined by leaving DPS samples at 5°C, 23°C, 37°C and 50°C for 0-4 weeks as listed in the table 7 A-D. Recovery was within our acceptance range of 70-130%, indicating that DPS samples can be safely stored at +5°C-37°C for at least 4 weeks without significant loss in recovery, and at 50°C for up to 2 weeks. Similar results were obtained for whole blood samples (data not shown)
