# Supplementary material for: A Simple Method to Quantitate IP-10 in Dried Blood and Plasma Spots
Source: PLoS One. 2012 Jun 27;7(6):e39228. doi: 10.1371/journal.pone.0039228 (PMC3384664; doi:10.1371/journal.pone.0039228)
Supplement: Table S8 — Stability of IP-10 in plasma stored at +5°C for up to 6 weeks. The stability of IP-10 in plasma was determined by leaving samples at 5°C for the weeks listed in the table. Samples were within our acceptance range of 70–130%, indicating that samples can be stored at +5°C for at least 6 weeks with no loss of signal. (DOCX) [file pone.0039228.s011.docx]

**Table S8 - Stability of IP-10 in plasma stored at +5°C for up to 6 weeks**

The stability of IP-10 in plasma was determined by leaving samples at 5°C for the weeks listed in the table. Samples were within our acceptance range of 70-130%, indicating that samples can be stored at +5°C for at least 6 weeks with no loss of signal
