# Supplementary material for: A Simple Method to Quantitate IP-10 in Dried Blood and Plasma Spots
Source: PLoS One. 2012 Jun 27;7(6):e39228. doi: 10.1371/journal.pone.0039228 (PMC3384664; doi:10.1371/journal.pone.0039228)
Supplement: Table S9 — Stability of IP-10 in plasma with storage at room temperature +23°C. The stability of IP-10 in a plasma sample stored at room temperature was assessed. %Recovery is calculated by comparing the value of the treated sample to the reference (0 days) sample ×100. Samples were within our acceptance range of 70–130%, indicating samples can be stored at room temperature for prolonged periods without significant loss in recovery. (DOCX) [file pone.0039228.s012.docx]

**Table S9. Stability of IP-10 in plasma with storage at room temperature +23°C**

The stability of IP-10 in a plasma sample stored at room temperature was assessed. %Recovery is calculated by comparing the value of the treated sample to the reference (0 days) sample x100. Samples were within our acceptance range of 70-130%, indicating samples can be stored at room temperature for prolonged periods without significant loss in recovery.
