# Supplementary material for: A Simple Method to Quantitate IP-10 in Dried Blood and Plasma Spots
Source: PLoS One. 2012 Jun 27;7(6):e39228. doi: 10.1371/journal.pone.0039228 (PMC3384664; doi:10.1371/journal.pone.0039228)
Supplement: Table S10 — Stability of IP-10 in plasma with repeated freeze thaw cycles. The ability of IP-10 in plasma to tolerate freeze-thaw cycles was assessed by freezing and thawing up to 10 times. Samples were thawed at room temperature and left for minimum 2 hours before refreezing. There was at least 24 hours between each freeze-thaw cycle. %Recovery is calculated by comparing the value of the treated sample to the freshly thawed control sample x100. Samples were within our acceptance range of 70–130%, indicating samples can undergo at least 10 freeze-thaw cycles without major loss in recovery. (DOCX) [file pone.0039228.s013.docx]

**Table S10. Stability of IP-10 in plasma with repeated freeze thaw cycles**

The ability of IP-10 in plasma to tolerate freeze-thaw cycles was assessed by freezing and thawing up to 10 times. Samples were thawed at room temperature and left for minimum 2 hours before refreezing. There was at least 24 hours between each freeze-thaw cycle. %Recovery is calculated by comparing the value of the treated sample to the freshly thawed control sample x100. Samples were within our acceptance range of 70-130%, indicating samples can undergo at least 10 freeze-thaw cycles without major loss in recovery
